# P16^INK4A^ is required for cisplatin resistance in cervical carcinoma SiHa cells

**DOI:** 10.3892/ol.2014.2814

**Published:** 2014-12-19

**Authors:** YUERAN LI, SONGSHU XIAO, LIU DAN, MIN XUE

**Affiliations:** Department of Obstetrics and Gynecology, The Third Xiangya Hospital of Central South University, Changsha, Hunan 410013, P.R. China

**Keywords:** P16^INK4A^, cervical carcinoma, cisplatin resistance, SiHa-cisplatin, cell cycle

## Abstract

Cervical cancer is the third most commonly diagnosed cancer worldwide and the fourth leading cause of cancer-related mortality in females worldwide, accounting for 10–15% of cancer-related mortalities. Cytological screening and DNA testing for high-risk human papillomavirus (HPV) types have markedly decreased the rates of cervical cancer in developed countries, however, for vulnerable populations without access to health care, cervical cancer remains a considerable problem. Chemotherapeutic agents such as cisplatin (DDP) are considered as first-line treatment for cervical carcinoma. Although initially patients often exhibit high responsiveness, the majority eventually develop DDP resistance. However, the mechanisms underlying this process remain unclear. Furthermore, patients with metastatic cancer and those exhibiting persistent or recurrent disease after platinum-based chemoradiotherapy have limited options and thus, non-platinum combination chemotherapy has been proposed as a strategy to circumvent platinum resistance, however, novel therapeutic strategies are required. In the present study, P16 expression was analyzed by quantitative-polymerase chain reaction and western blot analysis in SiHa and SiHa-DDP cells and the interaction between P16 and CDK4 was detected via co-immunoprecipitation. In addition, the proliferation and apoptosis rates of P16 knockdown SiHa-DDP cells were measured by MTT assay and Annexin V flow cytometry and the subsequent changes in cyclin D1 and pRb expression were analyzed by western blot analysis. In this study, a high level of P16^INK4A^ expression and its enhanced interaction with cyclin-dependent kinase-4 in cervical carcinoma DDP-resistance cells (SiHa-DDP) was identified, which was associated with the inactivation of phosphorylated retinoblastoma protein (pRb). Knockdown of P16^INK4A^ significantly induced cellular growth, when compared with the control cells, via the upregulation of pRb, and also promoted apoptosis following treatment with DDP. The results of this study indicated, for the first time, that P16^INK4A^ is required for DDP resistance in cervical carcinoma SiHa cells and, thus, these results may lead to the development of novel strategies for the treatment of chemoresistant cervical carcinoma.

## Introduction

Cervical cancer is the third most commonly diagnosed cancer and the fourth leading cause of cancer-related mortalities in females worldwide. Cervical cancer accounted for 9% (529,800) of all newly diagnosed cancer cases and 8% (275,100) of all cancer-related mortalities among females, worldwide in 2008 (1) and >85% of these cases and mortalities occurred in developing countries (1). According to the American Cancer Society, the five-year relative survival rate for uterine cervical cancer diagnosed between 2002 and 2008 in the USA was 69% (2). At present, standard treatment for cervical carcinoma treatment involves surgery, chemotherapy and radiotherapy. Combined chemotherapy using cisplatin (DDP) has been widely approved for the clinical treatment of cervical carcinoma. Furthermore, a marked decrease in mortality of patients has been observed with DDP-containing chemotherapy schemes in cervical carcinoma treatment (3,4). The efficacy of DPP appears to be a result of its ability to enter cells via multiple pathways and produce multiple DNA-platinum adducts, which initiate the apoptotic pathway, resulting in cell death (5). At present, DDP remains a front-line clinical therapy and constitutes part of the treatment regimen for patients with various types of cancer. However, drug resistance has become a major challenge associated with successful DDP treatment of cervical carcinoma. In addition, the molecular basis for resistance remains unclear.

DNA-platinum adducts may initiate a cellular self-defense system, resulting in significant epigenetic and/or genetic alternations. Previous studies have demonstrated that the mechanisms underlying DDP resistance are associated with supporting cell survival, including cell growth-promoting pathways, apoptosis, developmental pathways, DNA damage repair and endocytosis (6).

In human papillomavirus (HPV) infected cervical carcinoma, the HPV virus produces E6 and E7 proteins (7) that inactivate cyclin-dependent kinase inhibitors (CKI), including P16^INK4A^ and retinoblastoma protein (Rb), or cause the overexpression of cyclin D that releases active E2F, which induces cell cycle traversal (8). It has been reported that irreversible proliferation arrest is a drug-responsive program, able to influence the outcome of cancer chemotherapy (9). Certain CKIs, including P21, P27 and p53, regulate DDP resistance (8) by mediating the apoptotic pathway (10,11). However, the association between the cell cycle and DDP resistance in HPV-infected cervical carcinoma requires further study. The tumor suppressor and CKI, P16^INK4A^, which is usually overexpressed in cervical carcinoma, is not detectable in cervical carcinomas, which are predominantly sensitive to DDP (12,13), and the cyclin D-CDK4,6/p16/phosphorylated RB (pRb)/E2F cascade is altered in >80% of human tumors (14–17). However, the association between P16^INK4A^ and DDP resistance in cervical carcinoma remains unclear.

In the present study, the association between p16 and DDP resistance in cevical carcinoma was investigated. The mRNA and protein expression levels of P16, Cyclin D1 and pRb in SiHa and SiHa-DDP cell lines were detected, and p16 knock down of a SiHa-DDP cell line was performed to investigate the possible mechanism of DDP chemoresistance, which may lead to the development of novel treatment strategies for chemoresistant cervical carcinoma.

## Materials and methods

### Cell culture and transfection of shRNA

SiHa and SiHa-DPP cells were purchased from Professor Wang He (West China Second University Hospital, Sichuan, China). The SiHa cell lines were cultured in Dulbecco’s modified Eagle medium (DMEM; HyClone, Logan, UT, USA) supplemented with 10% fetal bovine serum (FBS; Hangzhou Sijiqing Biological Engineering Materials Co., Ltd., Hangzhou, China) and 50 U/ml penicillin and streptomycin (all from Beyotime Institute of Biotechnology, Haimen, China). Human normal oral keratinocytes were cultured in Oral Keratinocyte Medium (ScienCell Research Laboratories, Carlsbad, CA, USA) containing 5 ml oral keratinocyte growth supplement and 5 ml penicillin/streptomycin solution. Transfection was performed when cells had reached ~80% confluency. P16 shRNA (shP16) and the control shRNA (0.1 mg; mock) (Santa Cruz Biotechnology, Inc., Santa Cruz, CA, USA) vectors were transfected with lipofectamine LTX reagent with PLUS reagent (Invitrogen Life Technologies, Carlsbad, CA, USA). Following transfection, the cells were isolated using culture medium. Western blot analysis was then performed to determine the efficiency of p16 knockdown.

### mRNA expression analysis

Total RNA was isolated using TRIzol Reagent (Invitrogen Life Technologies) and reverse transcription was performed using the PrimeScript™ First Strand cDNA synthesis kit (Takara Biotechnology Co., Ltd., Dalian, China) according to the manufacturer’s instructions. cDNA was generated from 5 mg of total RNA, also according to the manufacturer’s instructions. Reverse transcription (RT) real-time quantitative polymerase chain reaction (qPCR) was performed to evaluate the expression levels of P16 mRNA in the SiHa cell lines. Quantitative PCR was performed using Takara SYBR Premix Ex Taq II (Takara Biotechnology Co., Ltd.) and an ABI PRISM® 7500 Sequence Detection System (Applied Biosystems, Foster City, CA, USA). The total reaction volume was 10 μl consisting of 5 μl 2× SYBR premix EX Taq™, 0.5 μl (10 μM) PCR forward primer, 0.5 μl (10 μM) PCR reverse primer, 0.5 μl cDNA and 3.5 μl dH_2_O. The sequences of the gene specific primers were as follows: Forward, 5′-CCTTTGGTTATCGCAAGCTG-3′; and reverse, 5′-CCCTGTAGGACCTTCGGTGA-3′ for P16; forward, 5′-CAAGGGTCATTATGGGTTAGGC-3′ and reverse, 5′-TTAGGTGTAGGGGAGGGGAGA-3′ for pRb; and forward, 5′-AGCCACATCGCTCAGACAC-3′ and reverse, 5′-GCCCAATACGACCAAATCC-3′ for GAPDH.

### Protein expression analysis

Proteins were extracted using cell lysis buffer (Beyotime Institute of Biotechnology) according to the manufacturer’s instructions. The protein concentration was quantified using the Enhanced BCA Protein Assay kit (Beyotime Institute of Biotechnology). For western blot analysis, equal amounts of total protein (20 μg) were boiled and separated by SDS-PAGE. Following electrophoresis, the protein was blotted onto a polyvinylidene fluoride membrane (EMD Millipore, Billerica, MA, USA) and blocked for 2 h at room temperature. The membranes were then incubated with human anti-mouse monoclonal p16 antibody (Sigma-Aldrich, St. Louis, MO, USA), human anti-mouse polyclonal pRb and β-actin antibodies (Cell Signaling Technology Inc., Danvers, MA, USA), human anti-rabbit monoclonal CDK4 antibody (Cell Signaling Technology, Inc.) overnight at 4°C. The membranes were then washed with Tris-buffered saline three times prior to incubation with horseradish peroxidase (HRP)-conjugated goat anti-rabbit CDK4 and HRP-conjugated goat anti-mouse p16, Rb and β-actin secondary antibodies (Beyotime Institute of Biotechnology) and visualized using an ECL substrate (Thermo Fisher Scientific, Waltham, MA, USA). The membranes were scanned using a myECL Imager (Thermo Fisher Scientific) and the relative level of protein expression was analyzed by ImageJ software (imagej.nih.gov/ij/).

### 3-(4,5-dimethyl-thiazol-2-yl)-2,5-diphenyltetrazolium bromide (MTT) assay

Cells proliferation was measured by MTT assay (Funakoshi Co., Tokyo, Japan) (20) in 96-well microculture plates (Thermo Fisher Scientific). The cells were seeded at a density of 1×10^4^ cells/well in 96-well plates in DMEM containing 10% FBS. Three duplicate wells were set up for each group and the experiment was peformed in triplicate. After 48 h incubation, 20 μl MTT solution (5 mg/ml) in phosphate-buffered saline (PBS; Beyotime Institute of Biotechnology), was added to each well for 4 h. The absorbance of each well was analyzed using an Infinite F50 microplate reader (Tecan, Männedorf, Switzerland) at a wavelength of 570 nm. Proliferation curves were plotted according to the optical density.

### Apoptosis assay

Apoptosis detection was carried out using Annexin V flow cytometry (Becton Dickinson, San Jose, CA, USA) as previously described (21). Cells were treated with 2 mM DDP or left untreated, and adherent cells were then harvested after 72 h and washed in cold PBS, centrifuged at 200 × g at 4°C for 5 min and resuspended in Annexin-binding buffer. Annexin V and propidium iodide (Invitrogen Life Technologies) were added to the cell resuspension and the stained cells were analyzed by flow cytometry using the S3 cell sorter (Bio-Rad, Hercules, CA, USA) at wavelengths of 530 and >575 nm.

### Statistical analysis

Statistical significance was determined using SPSS, version 16.0 (SPSS, Inc., Chicago, IL, USA). P<0.05 was considered to indicate a statistically significant difference. Data are expressed as the mean ± standard error of the mean.

## Results

### High P16^INK4A^ expression is associated with reduced cellular growth and apoptosis in SiHa-DPP cells

**Figure 1:**
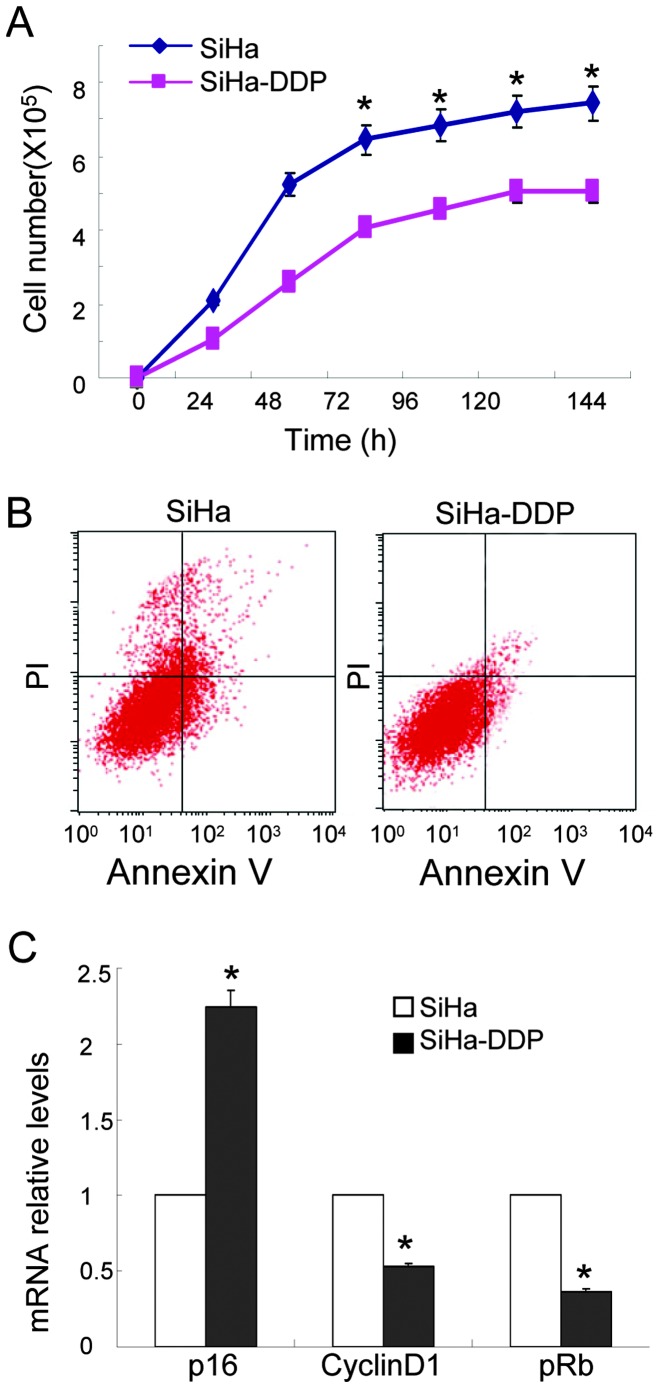
High P16^INK4A^ expression is associated with reduced cellular growth and apoptosis in SiHa-DDP cells. (A) MTT assay showed SiHa-DDP cells demonstrated a significant decrease in cellular growth when compared with the SiHa cells. Data are presented as the means ± SEM of values from three assays. (B) Cell death was analyzed by Annexin V/propidium iodide flow cytometry in SiHa-DDP and SiHa cells treated with 2 mM DDP. The numbers in the charts indicate less and later apoptosis in in SiHa-DDP treated by DDP. (C) Real time polymerase chain reaction indicated that P16 mRNA was markedly upregulated, while cyclin D1 and pRb were downregulated in SiHa-DDP cells when compared with SiHa cells. Data are presented as the mean ± SEM of values from three assays. ^*^P<0.05, compared with SiHa cells. DDP, cisplatin; MTT, 3-(4,5-dimethyl-thiazol-2-yl)-2,5-diphenyltetrazolium bromide; pRB, retinoblastoma protein.

The DDP-sensitive and -resistant cervical carcinoma cell lines were used to compare DDP responses. MTT assay and flow cytometry were performed to identify the characteristics of human cervical cancer cells (SiHa) and SiHa-DDP cells. As shown in Fig. 1A, reduced cellular growth was observed in the SiHa-DDP cells when compared with SiHa cells, and less apoptosis was observed in SiHa-DDP cells following treatment with DDP (Fig. 1B). qPCR demonstrated that the levels of P16^INK4A^ increased (Fig. 1C) while cyclin D1 and pRb levels were downregulated in SiHa-DDP cells when compared with SiHa cells. These results indicate that SiHa cells resistance to DDP was associated with high P16^INK4A^ expression levels.

### Levels of P16^INK4A^-CDK4 complex increase in SiHa-DPP cells with pRb inactivation

**Figure 2:**
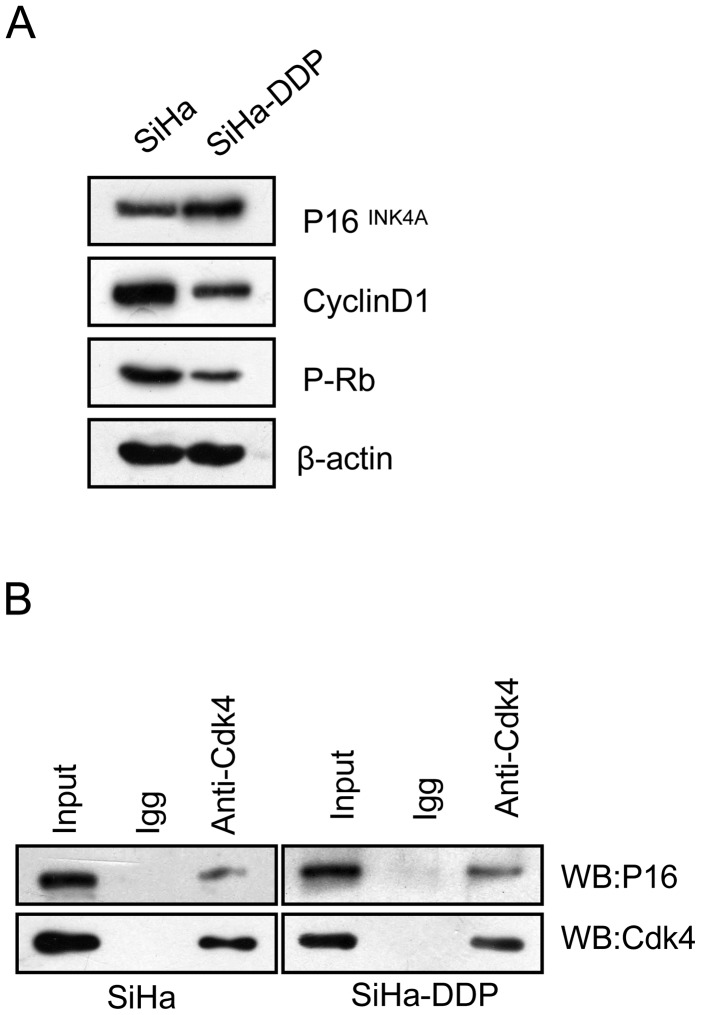
P16^INK4A^-CDK4 interaction is upregulated in SiHa-DDP cells with pRb inactivation. (A) Western blot analysis revealed the changes in P16, cyclin D1 and pRb protein expression, indicating an inverse correlation with P16 and cyclin D1, pRb in DDP-resistant SiHa-DPP cells. (B) Co-immunoprecipitation was conducted to evaluate the enhanced interaction between P16 and CDK4 in SiHa-DDP cells when compared with SiHa cells. DDP, cisplatin; Rb, retinoblastoma protein; pRb, phosphorylated retinoblastoma protein; CDK, cyclin-dependent kinase.

It has been hypothesized that the cyclin D1-CDK4-pRb pathway causes DDP resistance via cell cycle arrest (14,15). Therefore, the influence of P16 on the cyclin D1-CDK4-pRb pathway in SiHa-DPP cells was analyzed. Co-immunoprecipitation was performed to evaluate the interaction between P16^INK4A^ and CDK4 in SiHa-DDP cells and SiHa cells. The results showed that P16 was coprecipitated with CDK4 in both cell lines (Fig. 2A), and the interaction in SiHa-DDP was enhanced when compared with SiHa cells, indicating that the P16-CDK4 complex existed and may be involved in DDP-resistance. To investigate whether the enhanced levels of the P16-CDK4 complex contributes to cell cycle arrest, western blot analysis was performed to detect changes in pRb activity. As shown in Fig. 2B, pRb levels decreased concurrently with the upregulation of P16^INK4A^ protein levels in SiHa-DDP cells, indicating that P16 may compete with cyclin D1 to interact with CDK4, thus inhibiting pRb activation associated with DDP-resistance in SiHa-DDP cells.

### Knockdown of P16^INK4A^ induces growth and apoptosis in cells treated with DDP

**Figure 3:**
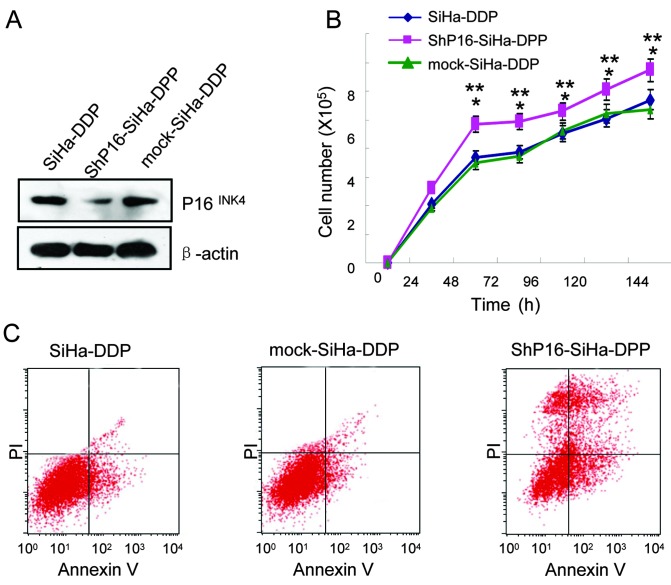
Knockdown of P16^INK4A^ induces growth and apoptosis in cells treated with DDP. (A) Western blot analysis showed that P16 proteins are markedly downregulated in shP16-transfected SiHa-DDP cells, but not in mock-transfected SiHa-DDP cells, when compared with SiHa-DDP cells. (B) 3-(4,5-dimethyl-thiazol-2-yl)-2,5-diphenyltetrazolium bromide assay showed shP16-transfected SiHa-DDP cells demonstrated a significant increase in cellular growth nor did mock-transfected SiHa-DDP cells compared with the SiHa-DDP cells. Data are presented as the mean ±SEM of values from three assays. ^*^P<0.05 vs. mock-transfected SiHa-DDP cells; **P<0.05 vs. SiHa-DDP cells. (C) Cell death was analyzed by Annexin V/propidium iodide flow cytometry in SiHa-DDP cells, shP16-transfected SiHa-DDP cells and mock-transfected SiHa-DDP cells treated with DDP (2 mM), The numbers in the charts indicate more and earlier apoptosis in shP16-transfected SiHa-DDP cells treated with DDP. DDP, cisplatin.

To investigate whether P16^INK4A^ regulates cellular growth and apoptosis in SiHa-DPP cells and contributes to DDP-resistance, shRNA and mock of P16^INK4A^ were transfected into SiHa-DPP cells. Western blot analysis was used to evaluate the protein levels of P16^INK4A^. It was indicated that P16^INK4A^ in shRNA-transfected cells was markedly downregulated when compared with mock-transfected cells (Fig. 3A). To investigate the proliferative effects in shP16-transfected cells, cellular growth was monitored for 144 h. The shP16-transfected SiHa-DPP cells exhibited a significant increase in cellular growth when compared with mock-transfected cells (P=0.05; Fig. 3B). Therefore, the effect of DDP treatment on the sensitivity of P16^INK4A^ knockdown cells to DNA damage was assessed. Flow cytometry revealed that shP16-transfected cells were more sensitive to DDP-induced apoptosis when compared with mock-transfected cells (Fig. 3C).

### P16^INK4A^ inactivates pRb in SiHa-DDP cells

**Figure 4:**
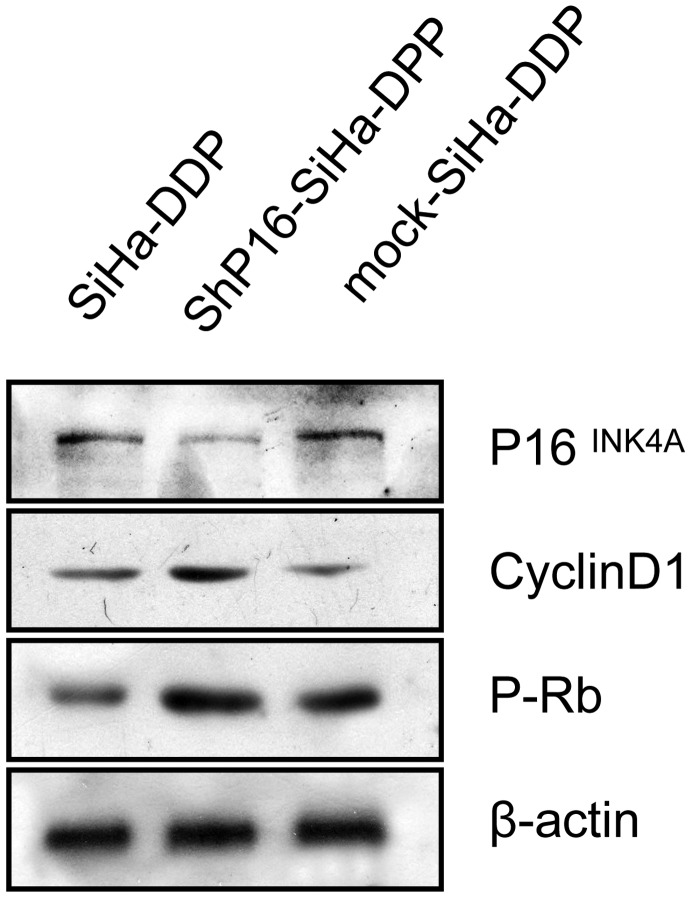
pRb and cyclin D1 levels are increased in shP16-transfected SiHa-DDP cells, western blot analysis revealed changes in P16, cyclin D1 and pRb protein levels, indicating that cyclin D1 and pRb are upregulated in shP16-transfected SiHa-DDP cells. pRb, phosphorylated retinoblastoma protein; DDP, cisplatin.

To identify the mechanism by which downregulated P16^INK4A^ influences proliferation, which contributes to DDP resistance, the protein expression of pRb and cyclin D1 in SiHa, mock-transfected SiHa-DDP cells and shP16-transfected SiHa-DDP cells was investigated. pRb and cyclin D1 were significantly increased in shP16-transfected SiHa-DDP cells (Fig. 4).

## Discussion

Rapidly proliferating cancer cells may be successfully killed by numerous chemotherapeutic drugs; however, several healthy proliferating cells are also damaged in the process, including hair follicle cells, intestinal cells and hematopoietic precursors. This nonselective killing of rapidly proliferating healthy cells often causes adverse effects in cancer patients. Several studies have indicated that CKIs may be used to protect normal cells from the toxicity caused by chemotherapy (8).

The cyclin D-CDK4,6/p16/pRb/E2F cascade has been found to be altered in a number of tumors (18). During the G1 phase of the cell cycle, Rb is inactivated by sequential phosphorylation events, which are mediated by various CKIs, including P16^INK4A^, which leads to the release of the E2F transcription factors and promotion of the cell cycle. It appears that the threshold level of the E2F1 protein, as well as the cell type, determine the function of the gene (17). DDP-resistant cells exhibited less and delayed growth when compared with non-resistant (siHa) cells (13). A reasonable explanation may be that the depressed growth of siHa-DDP cells was attributable to an elevated cellular p16 level, which caused proliferation arrest.

In the present study, SiHa human cervical carcinoma cells, which are known to be infected with HPV, were used (8). The results showed high P16^INK4A^ expression and its enhanced interaction with CDK4 in cervical carcinoma DDP-resistance cells (SiHa-DPP), which may cause irreversible proliferation arrest. Knockdown of P16^INK4A^ induced cells exiting the G1 phase into the S phase and promoted their proliferation, which enhanced DDP chemosensitivity as DDP targets cells in the S phase. Previous studies have revealed that the induction of P16^INK4A^, p21^Waf1^ or p27^Kip1^ lead to significant resistance to DDP-mediated cytotoxicity in the human cervical cancer cells, but not in the SiHa cells (19). This indicated that high expression of p16^INK4A^ in SiHa-DDP cells was required for DDP resistance, but did not cause it, implying that additional factors coordinated with P16^INK4A^, leading to DDP resistance in HPV-infected cervical carcinoma cells. Therefore, future studies are required to confirm the identity of such factors.

CKI-mediated resistance to chemotherapy may be a useful approach to protect normal cells from chemotherapy-induced toxicity in patients with pRb pathway-impaired cancer. Our results provided the first evidence that P16^INK4A^ regulated DDP resistance in cervical carcinoma SiHa cells, which may lead to the development of novel strategies for the treatment of chemoresistant cervical carcinoma.
